# The complete chloroplast genome of *Castanopsis sieboldii* (Makino) Hatus (Fagaceae)

**DOI:** 10.1080/23802359.2021.1966339

**Published:** 2021-08-24

**Authors:** Jongsun Park, Hong Xi, Janghyuk Son, Hyun Tak Shin, Hyunmi Kang, Seokgon Park

**Affiliations:** a InfoBoss Inc, Seoul, Republic of Korea; b InfoBoss Research Center, Seoul, Republic of Korea; c DMZ Botanic Garden, Korea National Arboretum, Yanggu, South Korea; d Department of Landscape Architecture, Mokpo National University, Muan, Republic of Korea; e Division of Forest Resources and Landscape Architecture, Sunchon National University, Sunchoen, Republic of Korea

**Keywords:** Chloroplast genome, *Castanopsis sieboldii*, interspecific variation, phylogenetic analysis, Fagaceae

## Abstract

*Castanopsis sieboldii* (Makino) Hatus is an evergreen tree that distributes in Eastern Asia including Islands of Korea and Japan. The chloroplast genome of *C. sieboldii* was successfully sequenced. Its length is 160,705 bp long (GC ratio is 36.8%) and has four subregions: 90,821 bp of large single copy (34.6%) and 19,014 bp of small single copy (30.8%) regions are separated by 25,075 bp of inverted repeat (42.8%) regions including 134 genes (89 protein-coding genes, eight rRNAs, and 37 tRNAs). Interspecific variations of *Castanopsis* are at a moderate level in comparison to those of the other genera. Phylogenetic trees show that *C. sieboldii* chloroplast genome was clustered with the other two *Castanopsis* species.

*Castanopsis sieboldii* (Makino) Hatus, belonging to Fagaceae, is a species of an evergreen tree that distributes in subtropical Eastern Asia, including Islands in Western and Southern Korea (Park, An, et al. 2020) and Honshu, Shikoku, and Kyushu Islands in Japan (Yamazaki 1987). This species is considered as a climax species in an evergreen forest (Yamanaka 1966). The genetic diversity of *C. sieboldii* in Japan is relatively high (Aoki et al. 2014): one of the major reasons is that they have been isolated in islands. It is one of hot topics to understand the origin of the island species based on chloroplast genomes, such as founder effects (Del Valle et al. 2019), multiple introductions in the islands (Nock et al. 2019), or the origin of endemic species in the islands (Yang et al. 2018; Park, Bae, et al. 2021). To understand its genetic diversity of *C. sieboldii* in Korea, as the first step, we completed the first chloroplast genome of *C. sibboldii* from the sample isolated in one of the islands in Korea, Oenalodo Island.

Total DNA of *C. sieboldii* collected at Singeum-gil, Bongnae-myeon, Goheung-gun, Jeollanam-do, Republic of Korea (34°28'18.58′'N, 127°28'04.41′'E) was extracted from fresh fruits with a DNeasy Plant Mini Kit (QIAGEN, Hilden, Germany). The voucher was deposited in the InfoBoss Cyber Herbarium (IN; http://herbarium.infoboss.co.kr/; Voucher number: IB-01087; Contact: Suhyeon Park; shpark817@infoboss.co.kr). Genome sequencing was conducted using NovaSeq6000 at Macrogen Inc., Korea, and *de novo* assembly and sequence confirmation were done by Velvet v1.2.10 (Zerbino and Birney 2008), GapCloser v1.12 (Zhao et al. 2011), BWA v0.7.17 (Li 2013), and SAMtools v1.9 (Li et al. 2009) in the Genome Information System (GeIS; http://geis.infoboss.co.kr/) which has been utilized in the previous organelle genomic studies (Park, Kim, Xi, Nho, et al. 2019; Park, Park, Kim, et al. 2019; Park, Yun, Oh, et al. 2019; Park, Lee, et al. 2021; Park, Min, et al. 2021; Joo et al. 2020; Park and Oh 2020; Kim et al. 2021). Geneious Prime^®^ v2020.2.4 (Biomatters Ltd., Auckland, New Zealand) was used for chloroplast genome annotation based on *Castanopsis fargesii* chloroplast (NC_047230; Ye et al. 2019).

The chloroplast genome of *C. sieboldii* (MZ028444) is 160,705 bp (GC ratio is 36.8%) and has four subregions: 90,821 bp of large single copy (34.6%) and 19,014 bp of small single copy (SSC; 30.8%) regions are separated by 25,705 bp of the inverted repeat (IR; 42.8%). It contains 134 genes (89 protein-coding genes, eight rRNAs, and 37 tRNAs); 20 genes (nine protein-coding genes, four rRNAs, and seven tRNAs) are duplicated in the IR regions.

Based on pair-wise alignments against chloroplast genomes of *C. fargesii* and *C. concinna*, distributed in Southern China (Sun et al. 2014; Daniel Hinsinger and Sergej Strijk 2017), 520 single nucleotide polymorphisms (SNPs) and 144 insertion and deletion (INDEL) regions covering 709 bp and 220 SNPs and 125 INDEL regions covering 533 bp were identified, respectively. Numbers of these interspecific variations are fewer in number than those of the four species of *Viburnum*, displaying 944 to 1295 SNPs and 1697-bp to 3080-bp INDELs (Park, Xi, et al. 2020), and *Potentilla micrantha* and *Potentilla centigrana* (1570 SNPs and 3,451-bp INDELs; Ferrarini et al. 2013; Park et al. 2019), and *Potentilla freyniana* and *Potentilla chinensis* (1236 SNPs and 2295-bp INDELs; Park et al. 2019; Dang et al. 2020). Moreover, they are similar or higher than the numbers of intraspecific variations identified from the samples between Korea and China (Heo et al. 2019; Oh et al. 2019a, 2019b; Park, Kim, Xi, Oh, et al. 2019; Park, Suh, et al. 2020; Heo et al. 2020; Oh and Park 2020). These results indicate that the numbers of interspecific variations identified from the three *Castanopsis* species are at a moderate level.

Thirteen Fagaceae chloroplast genomes including one outgroup species, *Betula platyphylla*, were used for constructing bootstrapped Maximum-Likelihood (ML), Neighbor-joining (NJ), and Bayesian Inference (BI) phylogenic trees using MEGA X (Kumar et al. 2018) and MrBayes v3.2.6 (Ronquist et al. 2012), respectively, after aligning whole chloroplast genomes by MAFFT v7.450 (Katoh and Standley 2013). A heuristic search was used with nearest-neighbor interchange branch swapping, the Tamura-Nei model, and uniform rates among sites to construct ML and NJ phylogenetic trees with default values for other options using MEGA X. Bootstrap analyses with 1000 and 10,000 pseudoreplicates were conducted for ML and NJ trees, respectively. The GTR model with gamma rates was used as a molecular model and Markov-chain Monte Carlo algorithm was employed for 1,100,000 generations, sampling trees every 200 generations, with four chains running simultaneously for BI tree. Three phylogenetic trees display that three *Castanopsis* species are clustered in one clade and are congruent to each other with high supportive values of ML, NJ, and BI (Figure 1). In addition, the topology of *Castanopsis*, *Castanea*, *Quercus*, and *Fagus* genera in the phylogenetic tree is congruent to the previous phylogenetic and morphological study (Manos et al. 2008). Taken together, our chloroplast genome is useful to investigate phylogenetic relationships of *C. sieboldii* as well as its genetic diversities along with geographical distribution.

**Figure 1.**
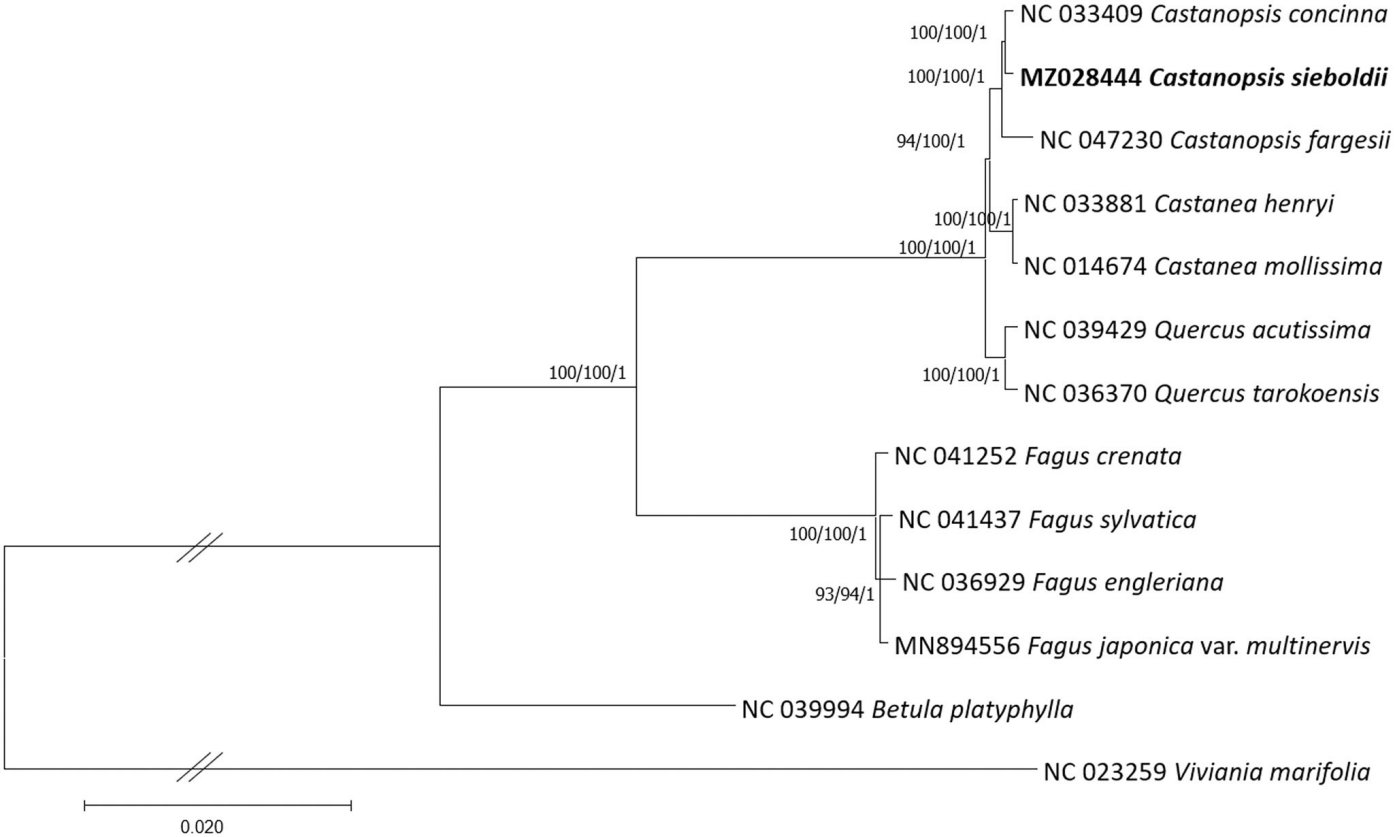
Maximum-Likelihood (bootstrap repeat is 1000), Neighbor-joining (bootstrap repeat is 10,000), and Bayesian inference (Number of generations is 1,100,000) phylogenetic trees of Fagaceae thirteen chloroplast genomes. Phylogenetic tree was drawn based on Maximum-Likelihood tree. The numbers above branches indicate bootstrap support values of Maximum-Likelihood, Neighbor-joining, and Bayesian inference phylogenetic trees, respectively.

## Data Availability

Chloroplast genome sequence can be accessed via accession number of MZ028444 in GenBank of NCBI at https://www.ncbi.nlm.nih.gov. The associated BioProject, SRA, and Bio-Sample numbers are PRJNA724746, SAMN18857600, and SRR14316975, respectively.
